# Identification of the osteoarthritis signature gene PDK1 by machine learning and its regulatory mechanisms on chondrocyte autophagy and apoptosis

**DOI:** 10.3389/fimmu.2022.1072526

**Published:** 2023-01-06

**Authors:** Jinzhi Meng, Huawei Du, Haiyuan Lv, Jinfeng Lu, Jia Li, Jun Yao

**Affiliations:** ^1^ Bone and Joint Surgery, The First Affiliated Hospital of Guangxi Medical University, Nanning, China; ^2^ Department of Pathology, The First Affiliated Hospital of Guangxi Medical University, Nanning, China

**Keywords:** osteoarthritis, PDK1, signature gene, autophagy, apoptosis

## Abstract

**Background:**

Osteoarthritis (OA) is a degenerative joint disease frequently diagnosed in the elderly and middle-aged population. However, its specific pathogenesis has not been clarified. This study aimed to identify biomarkers for OA diagnosis and elucidate their potential mechanisms for restoring OA-dysregulated autophagy and inhibiting chondrocyte apoptosis *in vitro*.

**Material and methods:**

Two publicly available transcriptomic mRNA OA-related datasets (GSE10575 and GSE51588) were explored for biomarker identification by least absolute shrinkage and selection operator (LASSO) regression, weighted gene co-expression network analysis (WGCNA), and support vector machine recursive feature elimination (SVM-RFE). We applied the GSE32317 and GSE55457 cohorts to validate the markers’ efficacy for diagnosis. The connections of markers to chondrocyte autophagy and apoptosis in OA were also comprehensively explored *in vitro* using molecular biology approaches, including qRT-PCR and Western blot.

**Results:**

We identified 286 differentially expressed genes (DEGs). These DEGs were enriched in the ECM-receptor interaction and PI3K/AKT signaling pathway. After external cohort validation and protein-protein interaction (PPI) network construction, PDK1 was finally identified as a diagnostic marker for OA. The pharmacological properties of BX795-downregulated PDK1 expression inhibited LPS-induced chondrocyte inflammation and apoptosis and rescued OA-dysregulated autophagy. Additionally, the phosphorylation of the mediators associated with the MAPK and PI3K/AKT pathways was significantly downregulated, indicating the regulatory function of PDK1 in apoptosis and autophagy *via* MAPK and PI3K/AKT-associated signaling pathways in chondrocytes. A significantly positive association between the PDK1 expression and Neutrophils, Eosinophils, Plasma cells, and activated CD4 memory T cells, as well as an evident negative correlation between T cells follicular helper and CD4 naive T cells, were detected in the immune cell infiltration analysis.

**Conclusions:**

PDK1 can be used as a diagnostic marker for OA. Inhibition of its expression can rescue OA-dysregulated autophagy and inhibit apoptosis by reducing the phosphorylation of PI3K/AKT and MAPK signaling pathways.

## 1 Introduction

Osteoarthritis (OA) is a degenerative joint disease and the most common joint disorder affecting the quality of life of older adults (1). Massive loss of the cartilage extracellular matrix leads to cartilage destruction and fibrous tissue growth, representing the main pathological features of OA (2, 3). Currently, it is possible to reduce OA symptoms by increasing the lubrication of the joint with some medications. However, due to its complex pathology, OA treatment strategies are unavailable. Increasing studies have shown that inflammatory storms during OA development cause apoptosis and autophagy dysregulation in chondrocytes, resulting in a decreased ability of cells to clear damaged organelles and incomplete proteins (4–6). Therefore, finding precise targets that regulate apoptosis and autophagy in OA chondrocytes is of great clinical importance.

Increasing evidence has shown that OA progression occurs along with autophagy attenuation in cells (7). As a protein degradation system in cells, autophagy protects cells from abnormal physiological conditions, such as hypoalimentation, endoplasmic reticulum stress (ERS), and hypoxia, by phagocytosis of dysfunctional organelles and incomplete proteins (8, 9). Attenuating autophagy in OA causes aggregation of different molecular proteins, leading to functional defects, cell degeneration, and eventually apoptosis. Currently, inhibiting chondrocyte apoptosis by activating and repairing dysregulated autophagy has become an interesting strategy for OA (10). Additionally, autophagic cell function is tightly mediated by signaling pathways, including MAPK- and PI3K/AKT-associated pathways.

Recently, gene microarray technology and bioinformatics have allowed the study of diseases in multiple dimensions, from transcriptomes and epigenetic changes to cellular mutations and copy number variations, to identify driver genes influencing disease progression. Bioinformatics is an emerging discipline that combines information technology and molecular biology. It is also an important tool for investigating the mechanisms underlying various diseases. Although many studies have explored OA diagnostic markers, their diagnostic value has not been well validated. Herein, we found and validated OA signature genes in GSE10575, GSE51588, GSE32317, and GSE55457 cohorts using bioinformatics tools. Additionally, the specific mechanisms between the identified OA signature genes in regulating cellular autophagy and apoptosis were further explored using *in vitro* experiments. The association of OA signature genes with the infiltration of 22 immune cells was also evaluated using the CIBERSORT (11, 12) algorithm and correlation analysis.

## 2 Materials and methods

### 2.1 Data acquisition and analysis

The transcriptome (GSE10575; GSE51588; GSE32317; GSE55457) of OA and normal cartilage tissues used for raw analysis were downloaded from the Gene Expression Omnibus (GEO, https://www.ncbi.nlm.nih.gov/geo/) platform. To reduce the error bias of individual samples, we used the “limma” and “SVA” R packages to combine and correct the GSE10575 and GSE51588 datasets as the training cohort for screening OA signature genes. We collected 62 samples, including 16 normal cartilage and 46 OA samples. Two independent datasets, GSE32317 and GSE55457, were used as validation cohorts. GSE32317 comprises early- and end-stage OA, and the GSE55457 dataset contained 10 normal and 23 OA samples. Additionally, differentially expressed genes (DEGs) were screened under the criterion of |log2 FC| ≥ 1 and FDR < 0.05 using the “limma” R package.

### 2.2 Molecular pathway and biological function enrichment analyses

We used KEGG analysis to detect the signaling pathways associated with DEGs in OA. Similarly, we conducted GO functional enrichment analysis to explore the biological functions of identified DEGs, including molecular functions (MFs), cellular components (CCs), and biological processes (BPs). To investigate the signaling pathways and biological functions activated in OA, we conducted Gene Set Enrichment Analysis (GSEA) (13).

### 2.3 Screening for signature genes

Three machine learning algorithms were used to identify significant markers to predict OA status. The LASSO regression analysis allows variable selection and complexity regularization to obtain accurate predictors. We performed the LASSO regression to identify genes with OA diagnostic significance using the “glmnet” R package. The SVM algorithm is based on supervised learning and binary data classification and has been widely used for classifying and subclassifying disease genomes (14). SVM’s powerful classification capabilities in disease genome learning can help discover new biomarkers and understand disease driver genes (15). The WGCNA (16) can identify gene modules with the highest relevance to diseases. Therefore, we used LASSO regression analysis, WGCNA, and the SVM-RFE algorithm to screen diagnostic genes in OA and the intersection of the three algorithms for further validation.

### 2.4 Validating the diagnostic value of OA signature genes

To evaluate the diagnostic accuracy of these signature genes for OA, receiver operating characteristics (ROC) curves were generated using the mRNA expression datasets from 16 normal cartilage and 46 OA samples of the training cohort. The area under the ROC curve (AUC) can accurately reflect the validity of the signature gene as a diagnostic marker. The results were further validated in the GSE32317 and GSE55457 cohorts. The screened diagnostic genes were also separately validated for differential expression in the two independent validation cohorts.

### 2.5 Protein-protein interaction network construction and correlation analysis

A PPI network was constructed using the online STRING database (https://cn.string-db.org/) (17), with the minimum required interaction score of 0.7, to explore PPIs between signature genes and the classical PI3K/AKT and MAPK inflammatory signaling pathway. The interrelationships between signature genes and inflammatory signaling pathways, chondrocyte autophagy, and apoptosis were further explored using the same approach to clarify the functions of signature genes in OA development.

### 2.6 Extraction and culture of chondrocytes

As previously described (18, 19), we collected knee cartilages from Sprague–Dawley (SD) rats (3-7 days old) under aseptic conditions, then extracted and cultured the chondrocytes *in vitro*. Briefly, after cutting into small pieces and 30-min digestion using trypsin (Solabio, China) at 37°C, the mixture was incubated with collagenase type II (1 mg/mL; Gibco), and high Glucose-containing DMEM medium (Gibco, USA) for 6 h. Chondrocytes were collected after centrifugation at 1500 rpm/3 min and cultured in FBS (10%; Gibco, USA) and penicillin/streptomycin (Solarbio, China) DMEM medium at 37°C. Third-generation chondrocytes were used for further studies. All experiments were approved by the Medical Ethics Committee of the First Affiliated Hospital of Guangxi Medical University (Approval number: 2022-E320-01).

### 2.7 Cytotoxicity and activity assay

We used the Cell Counting Kit-8 (CCK-8, C0037, Beyotime, China) assay to determine the safe concentration of the PDK1 inhibitor BX795 (HY-10514, MCE) and the PDK1 activator PS48 (GC13920, GLPBIO) on chondrocytes. Briefly, BX795 and PS48 were dissolved in dimethyl sulfoxide (DMSO, Beyotime, China) as a storage solution and stored at -20°C. To exclude the effects of DMSO on chondrocytes, storage solutions were diluted with phosphate-buffered saline (PBS, Solarbio, China) or DMEM medium during the experiment. The solubility of BX795 and PS48 was increased by heating to 37°C before the experiment. Third-generation chondrocytes were cultured in a 96-well plate (8×10^3^ cells per well) for 24 h, then intervened using BX795 (0.1-32 μM) and PS48 (2.5-320 μM) for two days. After adding 10 μL CCK-8 into each well of chondrocytes and 2-h culture, the absorbance values at 460 nm were recorded. To evaluate the viability of chondrocytes, we employed a live/dead cell staining kit (40747ES76, YEASEN, China). Briefly, chondrocytes were treated with BX795 (1 μM) or PS48 (5 μM) in 6-well plates (n = 8×10^4^ cells) after two days of incubation. PBS was rinsed clean of cell culture medium, then incubated with 4.5-μM propidium iodide (PI) and 2-μM Calcein-AM solution at 37°C for 30 min in the dark. After washing with PBS, a fluorescence imaging microscope (Olympus, BX53) was used to view and record the results.

### 2.8 Safranin-O staining

Third-generation primary chondrocytes were cultured in 6-well plates (8×10^4^ cells/well) for 48 h. The medium was aspirated and washed 3 times with PBS, followed by 4% paraformaldehyde (POM, Biosharp, China) soaking for 15-30 min. Finally, they were incubated with 0.2 mg/mL of L safranin-O staining solution (Solarbio, China) for 5 min in the dark.

### 2.9 RNA extraction and qRT-PCR

We used the RNAeasy™ Plus Animal RNA Isolation Kit with Spin Column (Beyotime, R0032, China) to extract total RNA from chondrocytes. The PrimeScript™ RT reagent Kit with gDNA Eraser (Takara, China) was used to reversibly transcribe 1 mg of the extracted RNA into cDNA. The expressions of PDK1, apoptosis-related genes (Bcl-2, Bax, and Casp-3), and autophagy-related genes (ATG7, LC3 I/II, and Beclin-1) in chondrocytes were measured by qRT-PCR. Primer sequences were designed according to the GenBank database and are shown in **Table 1**. A total reaction volume of 20 μL mix containing 10 μL SYBR Premix Ex Taq mixture (Thermo Fisher Scientific, USA), 0.8 μL of each primer, 3.4 μL sterile distilled water, and 5 μL cDNA was prepared before amplification. Target mRNA levels were compared to the control and normalized to GAPDH by the 2^-ΔΔCt^ method.

**Table 1:** Primer sequences used in this study.

Gene name	Forward primer (5′ to 3′)	Reverse primer (5′ to 3′)
GAPDH	AGTGCCAGCCTCGTCTCATA	GGTAACCAGGCGTCCGATAC
PDK1	CTTAGAGGGCTACGGGACGGATG	TCGTGGTTGGTTCTGTAATGCTTCC
BAX	AGACACCTGAGCTGACCTTGGAG	TTCATCGCCAATTCGCCTGAGAC
BCL-2	TGGAGAGCGTCAACAGGGAGATG	GTGCAGATGCCGGTTCAGGTAC
CASP-3	GCGGTATTGAGACAGACAGTGGAAC	AACCATGACCCGTCCCTTGAATTTC
ATG7	GATGGTGAACCTCAGCGGATGTATG	CAGCAGCAGGCACTTGACAGAC
LC3 I/II	GAGCGAGTTGGTCAAGATCATCCG	GATGTCAGCGATGGGTGTGGATAC
Beclin-1	TCAAGATCCTGGACCGAGTGACC	CTCCTCTCCTGAGTTAGCCTCTTCC

### 2.10 Western blot

Total chondrocyte protein was extracted using a 1% phosphatase inhibitor cocktail (CW2383, CWBIO), protease inhibitor cocktail (HY-K0010, MCE), and phenylmethanesulfonyl fluoride (PMSF, BOSTER, China)-contained RIPA lysis buffer (Beyotime, China). After determining the concentration using a BCA protein assay (Beyotime, China) and mixing with loading buffer and 10-min boiling at 100°C, protein samples were separated by 10% polyacrylamide gels. After transferring the protein bands using polyvinylidene fluoride (PVDF) membranes, membranes were blocked with 5% nonfat milk and incubated for 2 h at room temperature. Subsequently, blocked membranes were incubated with primary antibodies against GAPDH (Abcam), PDK1(Thermo Fisher Scientific), Beclin-1(Proteintech, China), LC3 I/II (Abcam), Atg7 (ZENBIO, China), P38 (Abcam), p-P38 (Abcam), JNK (CST), p-JNK (CST), ERK (Abcam), p-ERK (Abcam), p‐PI3K (Abcam), PI3K (Abcam), p‐Akt (Abcam), and Akt (Abcam) at 4°C overnight with 1:1000 dilution. After three washes with PBST and 1 h of incubation at room temperature with Rabbit anti-Goat IgG (H+L) Secondary Antibody (Thermo Fisher Scientific), signals were visualized with enhanced chemiluminescence (ECL, Beyotime) and recorded by Odyssey Infrared Imaging System.

### 2.11 Immunohistochemical analysis and Tunel staining

The donors agreed to use human-derived cartilage samples, and this study was approved by the Medical Ethics Committee of the First Affiliated Hospital of Guangxi Medical University (Approval number: 2022-E320-01). Cartilage sample sections were pretreated with 3% hydrogen peroxide, blocked with 10% goat serum (Gibco), and probed with PDK1 (1:400) primary antibody overnight at 4°C. Unbound antibodies were washed away with PBS and incubated with IgG secondary antibody (1:300, Thermo Fisher Scientific) for 2 h at room temperature. Next, diaminobenzidine (DAB) substrates (Boster, China) were used to develop the positively stained areas, and sections were counterstained with hematoxylin. Tunel staining (Promega) was performed to determine the level of apoptosis in sectioned bone tissues. Finally, sections were observed using an upright microscope (Olympus).

### 2.12 Immune cell infiltration and correlation analysis

The CIBERSORT (http://cibersort.stanford.edu/) (20) algorithm was used to quantify the content of infiltrated immune cells between OA and normal cartilage. We used the “corrplot” R package to analyze and visualize the relationship of the 22 infiltrated immune cells. We applied the “vioplot” R package to represent the variations of infiltrated immune cells between normal and OA cartilage samples with violin plots. Spearman’s rank correlation was conducted to clarify the correlation between infiltrated immune cell levels and PDK1 expression in different samples. Finally, the “ggplot2” R package was applied to visualize the associations.

### 2.13 Statistical analyses

Bioinformatics analyses were implemented in R (x64 4.0.2) and RStudio. The results of *in vitro* experiments are presented as means ± standard deviations. One-way analysis of variance (ANOVA) was performed between groups, followed by Tukey’s and t-test to determine significant differences. The significance level was set at *p* < 0.05, and figure legends indicate sample sizes.

## 3 Results

### 3.1 Identification of DEGs and functional enrichment analysis

**Figure 1:**
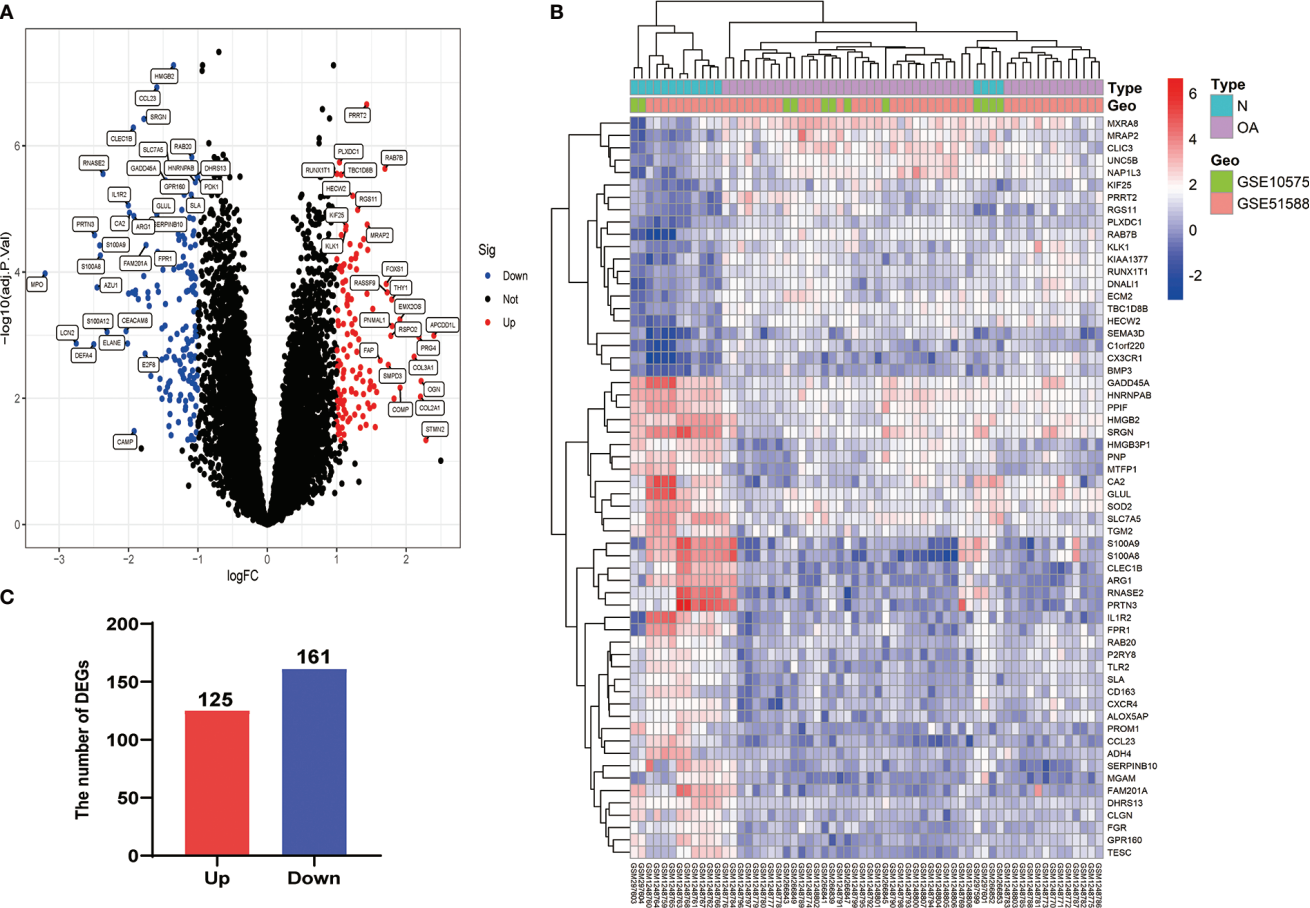
Visualization of DEG screening. **(A)** Volcano plot. Blue represents upregulated genes, red represents downregulated genes, and black represents genes with no difference. **(B)** Heatmap of the first 60 DEGs between different cohorts (GSE10575 and GSE51588) and sample types (Normal and OA). **(C)** Histogram of DEGs.

**Figure 2:**
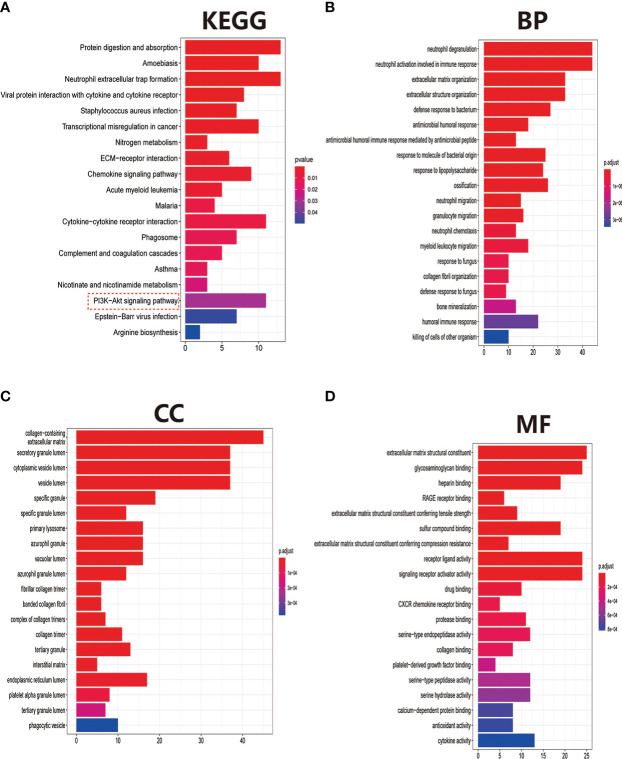
Pathway and biofunctional enrichment analyses. **(A)** KEGG pathway enrichment analysis. **(B-D)** GO functional enrichment analysis, including biological processes **(B)**, cellular components **(C)**, and molecular functions **(D)**.

After eliminating the batch effect of the two cohorts (GSE10575 and GSE51588), the ‘limma’ R package was used to calculate the merged cohorts’ differences. After screening, we identified 286 DEGs, 161 downregulated and 125 upregulated (**Figures 1A, C**). The distribution of DEGs among different cohorts and samples was visualized using a heatmap (**Figure 1B**). Meanwhile, we conducted KEGG and GO enrichment analyses to clarify the signaling regulatory pathways and biological functions of DEGs. The associations of the DEGs with the ECM-receptor interaction and the pathways associated with PI3K/AKT and chemokine were also observed (**Figure 2A**). Inhibition of the PI3K/AKT signaling pathway can promote autophagy activity and reduce the inflammatory response in chondrocytes (21, 22). We also found that DEGs were associated with neutrophil degranulation, humoral immune response, and neutrophil migration in the biological processes (BPs), suggesting that DEGs might have a regulatory relationship with immune regulation in OA (**Figure 2B**). Collagen fibers are important components of the extracellular matrix (ECM) of cartilage and play an important role in maintaining the growth and development of cartilage cells. In the cellular components (CCs) module, DGEs were significantly enriched on collagen fibers, banded collagen, and complex of collagen, indicating a close association between DGEs and cartilage development (**Figure 2C**). In terms of molecular functions (MFs), we also observed that DEGs were associated with extracellular matrix structural constituent conferring tensile strength, suggesting that DEGs were involved in the production of cartilage ECM and were associated with chondrocyte growth and development (**Figure 2D**). The degradation and abnormal metabolism of cartilage ECM are pathological features of OA development. The inhibition of cartilage ECM degradation and loss is an effective way to treat OA (23).

### 3.2 Gene set enrichment analysis

**Figure 3:**
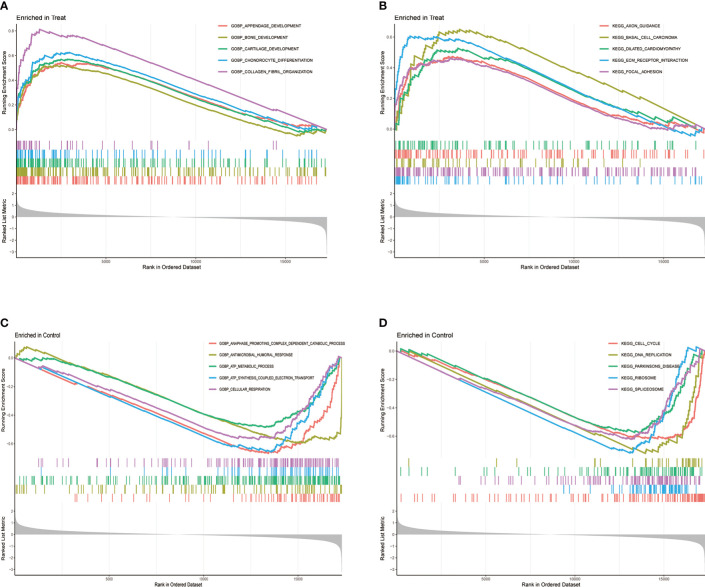
Gene Set Enrichment Analysis (GSEA) between treatment and control groups. **(A)** GO function and **(B)** KEGG signaling pathway enrichment in the treat group. **(C)** GO function and **(D)** KEGG signaling pathway enrichment in the control group.

The GSEA can be based on the entire gene expression dataset for biological functions and can more comprehensively and systematically reveal the different biological behavior and functional pathways between treatment and control groups. Herein, we found that the main biological behaviors enriched in the treatment group were related to skeletal system development, mainly bone development, cartilage development, chondrocyte differentiation, and collagen fibril organization (**Figure 3A**). Regarding the regulation of signaling pathways, the treatment group was mainly enriched in axon guidance, and ECM preceptor interaction (**Figure 3B**). Cartilage ECM plays a crucial role in cartilage development and differentiation. Increased cartilage ECM catabolism is a key factor in OA disease progression (24). The main enriched biological functions and signaling pathways in the control group were ATP syntheses coupled electron transport, cellular respiration (**Figure 3C**), cell cycle, and DNA replication (**Figure 3D**).

### 3.3 Identification and validation of OA signature genes

**Figure 4:**
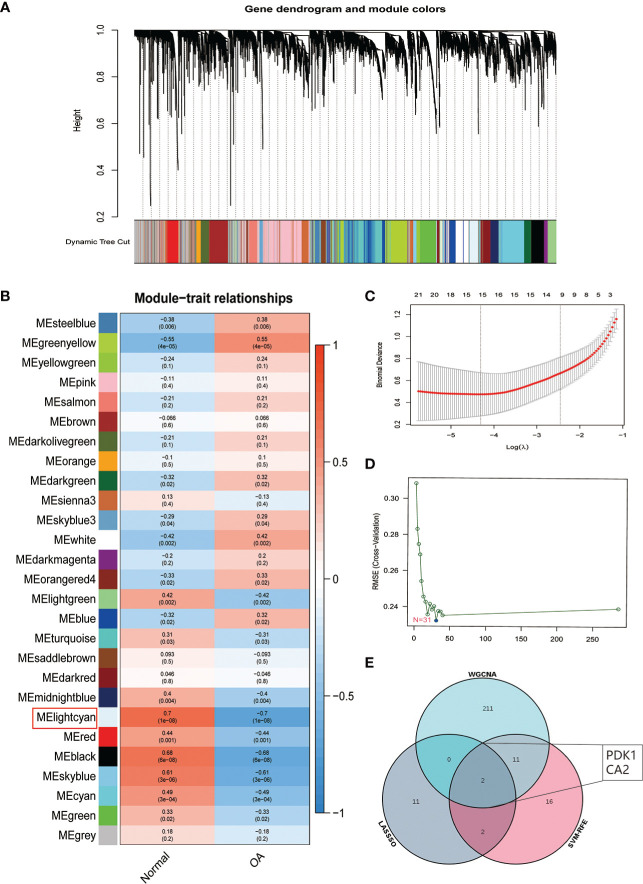
Identification of OA signature genes. **(A)** Module trait-related tree diagram in WGCNA. In the tree diagram, the top half is a hierarchical clustering diagram of genes, and the bottom half is a diagram of gene modules, with the top and bottom corresponding to each other. **(B)** Visualization of gene modules associated with OA. Each module is represented by a row and each trait by a column. The leftmost color block represents the module, the rightmost color bar represents the range of correlations, and the two middle columns represent the traits of normal cartilage and OA cartilage, respectively. In the middle heatmap section, the darker the color, the higher the correlation. Red indicates a positive correlation, blue indicates a negative correlation, and the number in each cell indicates the correlation and significance *p*-value. **(C)** The plot of OA disease signature genes was screened using the LASSO regression model. **(D)** OA disease signature genes identified by the SVM-RFE algorithm. **(E)** Venn diagram showing two signature genes (PDK1, CA2) crossed by the WGCNA, LASSO regression, and SVM-RFE algorithm.

**Figure 5:**
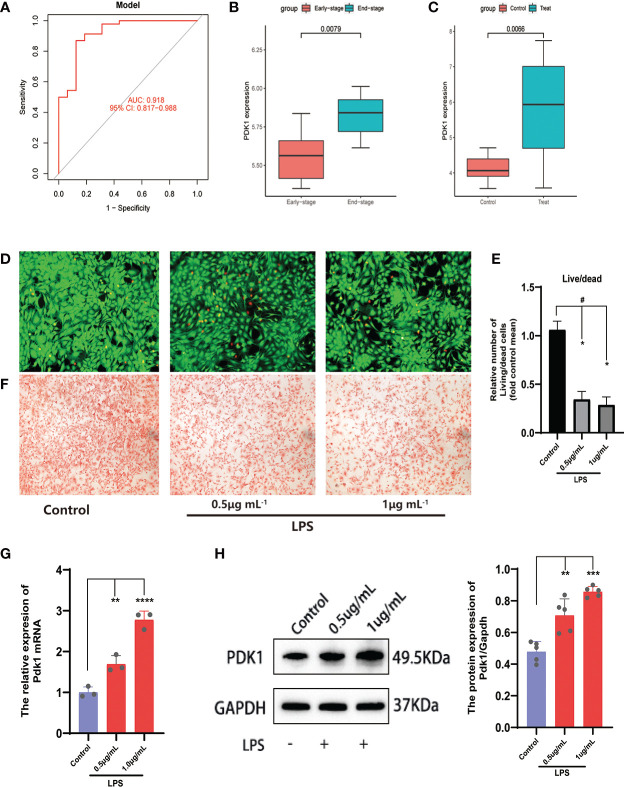
Analysis and validation of differential expression of the feature gene PDK1. **(A)** The ROC curve of the signature genes model for predicting disease. **(B, C)** Differential expression analysis of PDK1 in the **(B)** GSE55457and **(C)** GSE32317 cohorts. In the OA cell model, **(D)** Calcein-AM/PI staining was used to observe cell viability and **(E)** quantitative analysis. Chondrocytes in the control group were left untreated and were induced with 0.5 and 1 μg/mL LPS in the LPS group (means ± SD, n=3, *P<0.05, significantly different with control; ^#^ *P*<0.05, significantly different as indicated). **(F)** Safranin O for GAG production. **(G, H)** The relative expression of PDK1 mRNA **(G)** and protein expression levels **(H)** between OA and normal chondrocytes in the LPS group. Values are presented as means ± SD (n ≥ 3). ** *p* < 0.01; *** p < 0.001; **** *p* < 0.0001, relative to the control group. OA: osteoarthritis; qRT‐PCR: quantitative real‐time polymerase chain reaction; SD: standard deviation.

We used three algorithms to identify OA signature genes more accurately: WGCNA, LASSO regression, and SVM-RFE. WGCNA can classify and visualize genes into different modules according to their different relevance to the disease (**Figure 4A**). We identified the genes in the MElighcyan module as the most relevant gene set for OA based on the highest absolute value of correlation (**Figure 4B**). Thus, we selected the MElightcyan gene set with the highest correlation coefficient for subsequent analysis, containing 224 genes (**Table S1**). Besides, 15 genes were identified as OA diagnostic markers *via* LASSO regression (**Figure 4C**). Thirty-one OA signature genes were identified by the SVM-RFE algorithm (**Figure 4D**). Furthermore, the intersection of the feature genes obtained by the three algorithms was retrieved, and two (PDK1, CA2) were confirmed as OA diagnostic signature genes (**Figure 4E**). Next, we constructed ROC curves to evaluate the diagnostic ability of these two biomarkers to discriminate OA and control samples. The AUC was 0.898 for PDK1 and 0.856 for CA2 (Figure S1A). These results were further validated in the GSE32317 and GSE55457 cohorts. In the GSE32317 cohort, PDK1 and CA2 showed favorable diagnostic values with AUC of 0.875 and 0.903, respectively (**Figure S1B**). Similarly, in the GSE55457 cohort, PDK1 and CA2 also showed good diagnostic efficacy with AUC values of 0.796 and 0.661, respectively (**Figure S1C**). Furthermore, the model accuracy of the signature genes PDK1 and CA2 for predicting disease was further evaluated with an AUC of 0.918, indicating that the signature genes have a very credible value for predicting OA disease (**Figure 5A**).

Subsequently, we validated the differential expression of PDK1 and CA2 in OA using the GSE32317 and GSE55457 cohorts. PDK1 expression was higher in end-stage OA than in early-stage and higher in OA tissue than in normal cartilage (**Figures 5B, C**), suggesting that as OA disease progresses and the degree of inflammation increases, the expression level of PDK1 increases. This hypothesis was further confirmed by *in vitro* cellular experiments. We used different concentrations (0.5 and 1μg/mL) of LPS-induced OA chondrocyte models and characterized the survival of chondrocytes by calcein-AM/PI staining (**Figures 5D, E**). We observed that the growth activity of chondrocytes decreased with the increasing of LPS concentration, and the same result was observed in the safranin-o staining (**Figure 5F**) of the chondrocytes. The growth activity of the chondrocytes in the control group was significantly better than in the LPS group. Additionally, a positive correlation of the PDK1 expression with the dose of LPS was observed in both qRT-PCR (**Figure 5G**) and Western blot (**Figure 5H**). In contrast, there was no difference in CA2 expression between OA and normal cartilage (**Figure S1E**), except in early and end-stage OA samples (**Figure S1D**).

### 3.4 PDK1 was associated with the regulation of PI3K/AKT and MAPK pathways

**Figure 6:**
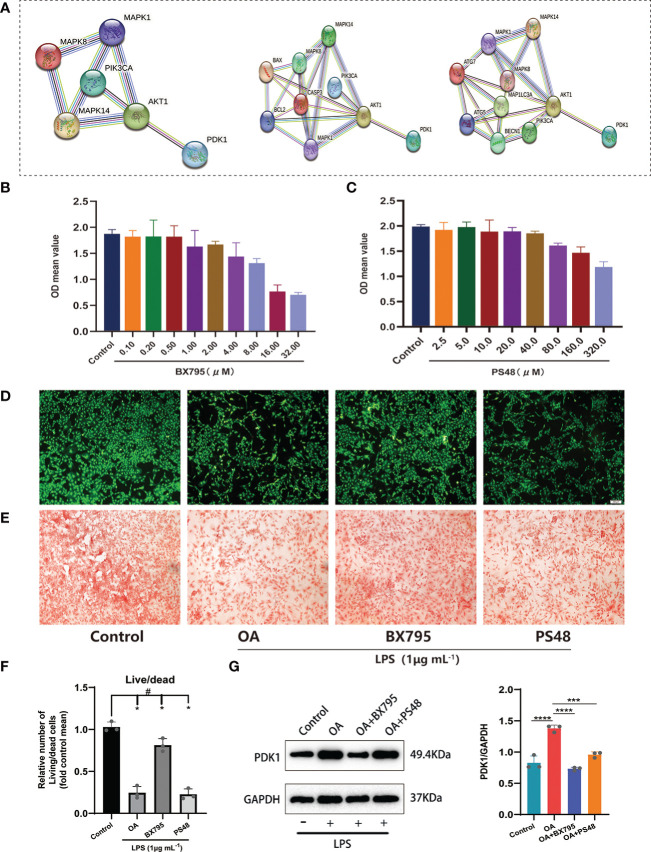
Exploring the potential signaling pathway of PDK1 involved in OA disease. **(A)** PPI network between PDK1 and the MAPK, PI3K/AKT signaling pathways, apoptosis, and autophagy-related mediators. Cytotoxic effect of different **(B)** BX795 (0.1-32 μM) and **(C)** PS48 (2.5-320 μM) concentrations on chondrocytes by the CCK-8 assay. **(D)** Calcein-AM/PI staining to detect the effects of BX795 (1 μM) and PS48 (5 μM) working concentrations on chondrocyte activity. Green represents viable cells, and red represents dead cells (scale bars = 200 μm.). **(E)** Safranin O staining was used for GAG production. Control: untreated chondrocytes; OA: chondrocytes cultured with 1 μg/mL LPS; BX795: LPS induction of chondrocytes for 24 h, then 1 μM of BX795 was added to continue the culture for 24 h; PS48: LPS induction of chondrocytes for 24 h, then 5μM of PS48 was added for culture for 24 h. **(F)** The quantitative analysis of Calcein-AM/PI staining (means ± SD, n=3, *P<0.05, significantly different with control; ^#^P<0.05, significantly different as indicated). **(G)** Wstern blot analysis verified that BX795 inhibited PDK1 protein expression and quantification (means ± SD, n=3, *** *p* < 0.001; **** *p* < 0.0001).

Based on the insights into the enrichment of DEGs in the PI3K/AKT signaling pathway (**Figure 2A**), we constructed PPI networks of diagnostic markers PDK1, CA2 with classical inflammatory signaling pathways (PI3K/AKT and MAPK), autophagy-related factors, and apoptosis-related factors to explore the potential relationship between diagnostic markers and inflammation regulation, autophagy, and apoptosis in OA (**Figure 6A**). Only PDK1 interacted with PI3K/AKT and MAPK pathways, autophagy-related factors, and apoptosis-related factors, suggesting that PDK1 might be involved in the autophagy and apoptosis of chondrocytes *via* PI3K/AKT and MAPK pathways. Therefore, we excluded CA2 from the subsequent experiments and used only PDK1. The CCK-8 kit was used to assess the potential cytotoxicity of the PDK1 inhibitor BX795 and the PDK1 activator PS48. Chondrocytes were cultured with different concentrations of BX795 (0.1-32 μM) and PS48 (2.5-320 μM). BX795 did not present cytotoxicity to chondrocytes between 0.1-1 μM compared to controls (**Figure 6B**), as well as PS48 between 2.5-10 μM (**Figure 6C**). Therefore, 1 μM of BX795 and 5 μM of PS48 were used for subsequent experiments.

### 3.5 LPS-induced chondrocytes and live/dead staining

Furthermore, we used 1 μg/mL LPS (Solarbio, China) to induce OA cell models *in vitro*. We divided the chondrocytes into four groups: (1) the control group, no treatment was given to the cells; (2) the OA group, chondrocytes were cultured with LPS (1 μg/mL); (3) OA+BX795 group, chondrocytes were induced by LPS for 24 h, then 1 μM of BX795 was added for culture for 24 h; (4) the OA+PS48 group, chondrocytes were induced by LPS for 24 h, then 5μM of PS48 for culture for 24 h. Our observations showed that most viable cells were found in the control group, followed by the BX795, OA, and PS48 groups (**Figures 6D, F**), and the same result was observed in the safranin-o staining (**Figure 6E**). Moreover, to understand the effect of PDK1 inhibitor BX795 and agonist PS48 on PDK1 protein expression, western blot was used to detect PDK1 protein expression between different groups, and the results showed that PDK1 protein expression in chondrocytes was decreased under the influence of BX795 (**Figure 6G**).

### 3.6 Inhibition of PDK1 can inhibit the activation of PI3K/AKT and MAPK inflammatory signaling pathways

**Figure 7:**
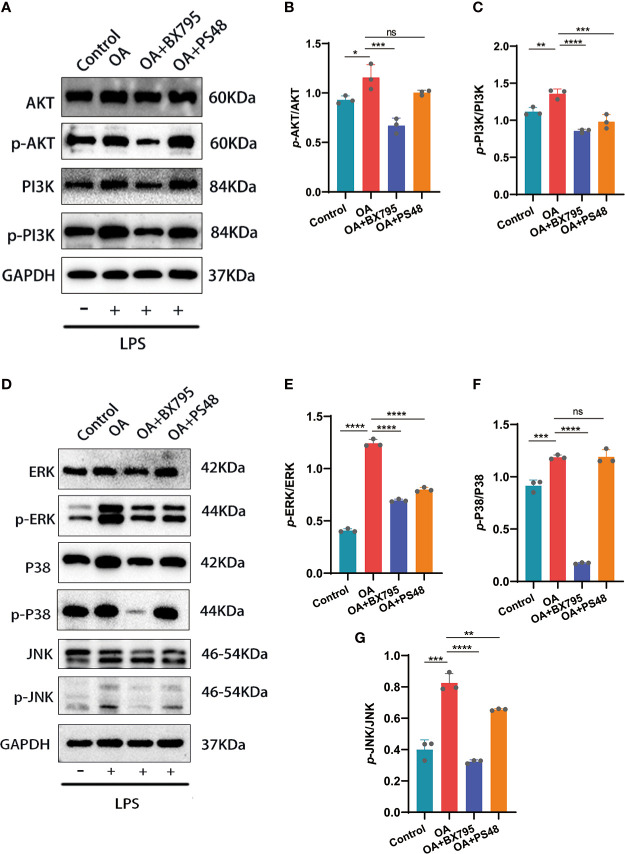
Western blot and quantitative analysis of the effects on MAPK and PI3K/AKT signaling pathways after targeted PDK1 downregulation. **(A-C)** Protein levels of mediators related to the PI3K/AKT signaling pathway by Western blot and quantification analysis. B: quantification analysis of *p*-AKT/AKT; C: quantification analysis of *p*-PI3K/PI3K. **(D-G)** Western blot and quantification analysis for the protein levels of MAPK signaling pathway mediators. E, F, and G: quantitative analysis of *p*-ERK/ERK, *p*-p38/p38, and *p*-JNK/JNK, respectively. Values are presented as means ± SD (n =3). * *p* < 0.05; ** *p*<0.01; *** *p* < 0.001; **** *p* < 0.0001; ns, no significance, relative to the OA group. OA, osteoarthritis; SD, standard deviation.

Through bioinformatic analysis and PPI construction, we have identified that PDK1 is involved in OA disease process and has protein interactions with mediators related to MAPK, PI3K/AKT signaling pathway. The phosphorylation of PI3K/AKT mediated by LPS was significantly attenuated after the PDK1 inhibitor was used in chondrocytes (**Figures 7A-C**). Similarly, we performed a Western blot analysis of the MAPK pathway and found that PDK1 downregulation using a PDK1 inhibitor significantly reduced the phosphorylation levels of MAPK inflammatory signaling pathway-related mediators (ERK, P38, and JNK) (**Figures 7D-G**). In summary, the phosphorylation of MAPK and PI3K/AKT inflammatory signaling pathways was attenuated by inhibiting PDK1 expression in OA cartilage, providing novel insights into inflammation inhibition and treatment in OA.

### 3.7 Inhibition of PDK1 in OA chondrocytes promotes autophagy and suppresses apoptosis

**Figure 8:**
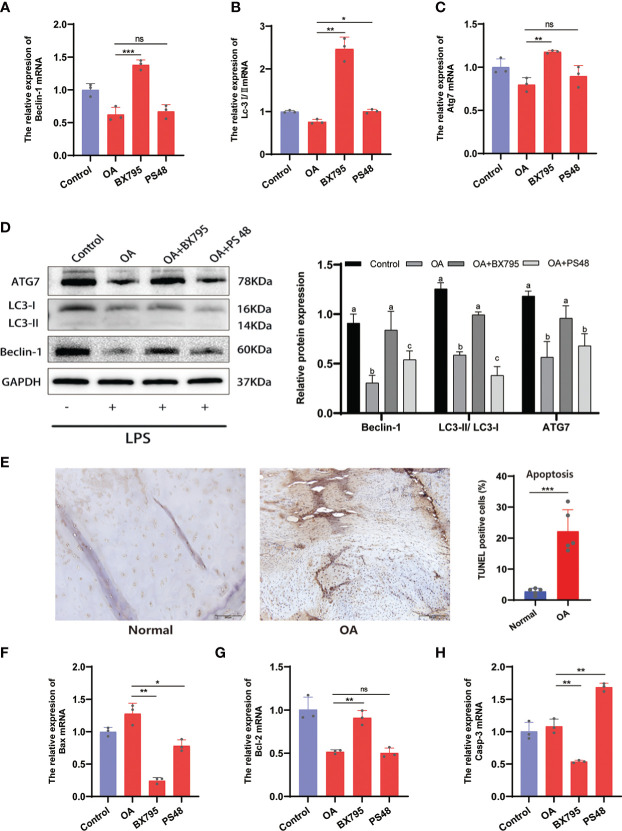
Inhibition of PDK1 expression restores OA-dysregulated autophagy and suppresses apoptosis. **(A-C)** Relative mRNA levels of Beclin-1, LC3I/II, and Atg7 by qRT-PCR. **(D)** The expression levels of autophagy-related proteins Beclin-1, LC3I/II, and Atg7 were detected by Western blot. In the quantitative plot, the bars with different letters are significantly different from each other, and the same letter represents no difference (*P*<0.05). **(E)** Tunel staining between OA and normal cartilage tissues revealed that apoptosis was significantly higher in the OA group than in the normal group (scale bars = 200 μm, n = 5). **(F-H)** Relative mRNA levels of Bax, Bcl-2, and caspase-3 (Casp-3) by qRT-PCR. Values are presented as means ± SD (n = 3). * p < 0.05; ** *p*<0.01; *** *p* < 0.001; ns, no significance, relative to the OA group. OA, osteoarthritis; SD, standard deviation.

The apoptosis and autophagy of the chondrocytes are significantly connected to OA progression. Autophagy is a cellular activity that maintains cellular homeostasis and cellular activity by scavenging free oxygen radicals and fragmented organelles (25). Restoration of dysregulated autophagy in OA can increase cartilage protection and attenuates cartilage matrix degradation. Conversely, increased chondrocyte apoptosis exacerbates the degradation of the cartilage ECM and OA (25, 26). Here, we have found that PDK1 interacted with apoptosis and autophagy in chondrocytes through the MAPK and PI3K/AKT signaling pathways (**Figure 6A**). The qRT-PCR results confirmed this hypothesis (**Figures 8A-C**). The mRNA expression of autophagy-related markers (Beclin-1, Lc3I/II, and Atg7) was elevated in chondrocytes with suppressed PDK1 expression by BX795 compared to the OA group. The same results were further validated at the protein expression level *via* western blot analysis (**Figure 8D**). We collected cartilage from OA patients with their corresponding adjacent normal cartilage for Tunel staining in the OA chondrocyte apoptosis analysis. We detected more apoptotic cells in the OA cartilage than in normal cartilage (**Figure 8E**), suggesting that inhibition of apoptosis in chondrocytes is a desirable option for treating OA progression. The qRT-PCR results revealed that PDK1 inhibition downregulated various proteins associated with cell apoptosis, such as Bax, Bcl-2, and Casp-3. These results indicated that PDK1 inhibition rescued OA chondrocyte apoptosis (**Figures 8F-H**).

### 3.8 Relationship between PDK1 and immune regulation in OA

**Figure 9:**
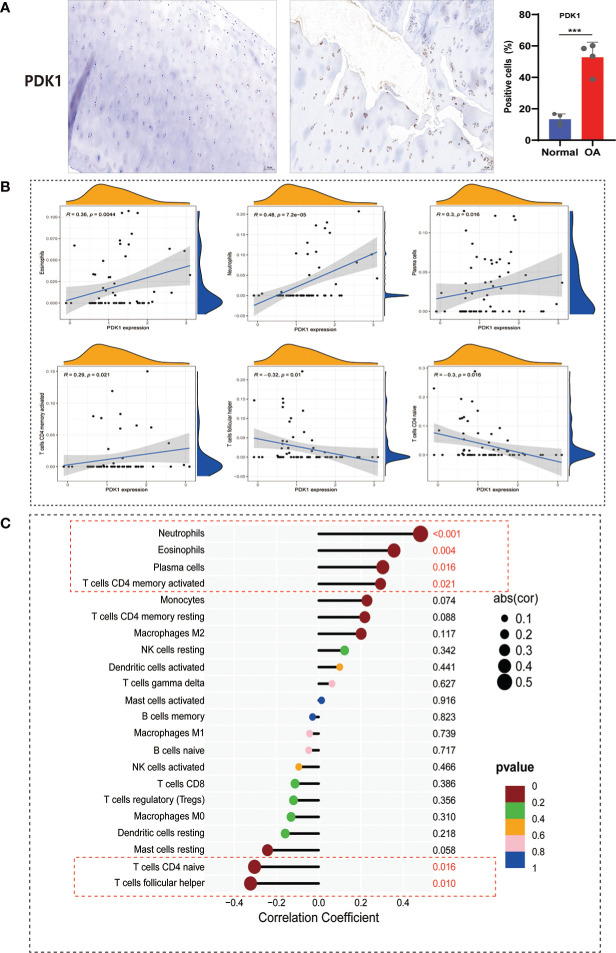
Correlation between PDK1 expression and immune cell abundance. **(A)** PDK1 immunohistochemical analysis between human normal cartilage and OA cartilage showed that PDK1 was highly expressed in OA tissues (means ± SD, n = 4,*** P < 0.001). **(B)** PDK1 expression levels were positively correlated with Neutrophils, Eosinophils, Plasma cells, and activated CD4 memory T cells and negatively associated with CD4 naive T cells and follicular helper T cells (*p* < 0.05). **(C)** Lollipop plot for the correlation between PDK1 and 22 immune cells. A *p* < 0.05 was considered statistically significant.

The immune system is divided into adaptive and innate immunity and is related to the body’s defense. Innate immune activation has been observed in cartilage damage in OA (27). To further understand the correlation between PDK1 and 22 immune cell infiltration in OA. First, we collected human cartilage tissues and used immunohistochemical analysis to confirm once again that PDK1 was overexpressed in OA cartilage tissues (**Figure 9A**). Next, we used the CIBERSORT algorithm to systematically analyze the association of PDK1 expression with the infiltration of 22 immune cells. The abundance of 22 immune cell infiltrates between normal and OA samples is shown in **Figure S2A** and **Figure S2B**. In addition, we observed differences in different types of immune cell infiltration between normal and OA samples, including T cells follicular helper, NK cells, Monocytes, Macrophages M1, Dendritic cells, Eosinophils, and Neutrophils (**Figure S2C**). PDK1 expression was positively associated with activated CD4 memory T cells (r = 0.29, *p* = 0.021), Plasma cells (r = 0.3, *p* = 0.016), Eosinophils (r = 0.36, *p* = 0.0044), and Neutrophils (r = 0.48, *p*<0.0001), while negatively associated with follicular helper T cells (r = -0.3, *p* = 0.016), and naïve CD4 T cells (r = -0.32, *p* = 0.01) (**Figure 9B**). The association of PDK1 with 22 immune cells can be visualized in **Figure 9C**.

## 4 Discussion

Current clinical treatment options for OA include conservative medication and joint replacement surgery. Drug therapy temporarily delays OA progression and does not reverse the patient’s disease state. Timely and early intervention is significant for OA treatment. Therefore, exploring useful diagnostic markers for early detection and timely treatment is of great clinical significance for OA prevention and therapy. However, early diagnostic tools for OA are still lacking in clinical care and are only captured by clinical imaging tools when the disease exhibits pathological features (28, 29). Therefore, in the present study, we systematically and comprehensively screened for the first-time disease signature genes for OA based on publicly available databases and conducted a series of *in vitro* cell experiments for validation and exploration. We detected 286 DEGs using differential analysis, and enrichments in the PI3K/AKT pathway were also observed (**Figure 2A**), suggesting that OA signature genes might regulate OA through the PI3K/AKT pathway. This hypothesis was supported by previous studies in which the PI3K/AKT inflammatory signaling pathway was involved in regulating OA (22, 30).

Moreover, we extended our exploration of the MAPK inflammatory signaling pathway and found that it was equally involved in the regulation of OA as PI3K/AKT (**Figure 6A**). Finally, we identified PDK1 as an OA disease signature gene using WGCNA, LASSO regression, SVM-RFE algorithm, and PPI network, which was comprehensively validated using the GSE32317, GSE55457 cohorts, and *in vitro* cellular experiments. Overall, we provided a new potential target for OA treatment and therapy.

Autophagy is a metabolic and innate immune process that maintains cellular self-stabilization and supports organelle renewal by removing broken proteins and degraded organelles (31). To avoid apoptosis and preserve cellular homeostasis, autophagy is frequently triggered during stressful situations such as hypoxia, malnutrition, and ROS generation (32–34). Unfortunately, the inflammatory microenvironment of OA disrupts autophagic activity and the energy balance of chondrocytes (35). Indeed, we observed significantly suppressed ATG7, LC3-I/II, and Beclin-1 expressions in chondrocytes after LPS induction, while this autophagic imbalance activity was rescued by PDK1inhibition (**Figures 8A-D**). As a pathological manifestation of OA, the apoptosis of chondrocytes is positively connected to the destruction severity of cartilage tissue and matrix depletion in OA (36, 37). The Tunel staining demonstrated that, compared to normal cartilage tissues, OA tissues exhibited significantly increased cell apoptosis (**Figure 8E**). Therefore, exploring a pathway that can inhibit cartilage apoptosis would bring new insights into the clinical treatment of OA. We found that PDK1 inhibition reduced BAX and Casp-3 expression, significantly associated with apoptosis, and enhanced BCL-2 expression, a BAX antagonist (**Figures 8F-H**). Thus, we speculated that, as an OA signature gene, PDK1 inhibition could repair the OA-dysregulated autophagy and interrupt the apoptotic program to play a cartilage-protective role.

As a significant member of the GAG protein kinase family, PDK1 functions in cells through various pathways, including PI3K/AKT and MAPK (38, 39). As pathways that significantly participate in pro-inflammatory responses, MAPK-related pathways are associated with OA pathogenesis and can provide cartilage protection by downregulating the MAPK signaling pathway (40, 41). Consistently, we found that PDK1 significantly participated in the mediation of the MAPK pathway, and PDK1 inhibition downregulated the phosphorylation of MAPK signaling pathway-related mediators (**Figures 7D-G**). Hence, we hypothesized that PDK1, as an upstream factor of the MAPK pathway, tightly participates in the regulation of inflammatory signaling in OA, and PDK1 inhibition could attenuate inflammation and restore OA-dysregulated autophagy. Additionally, the signaling pathway mediated by PI3K/AKT significantly regulates inflammation and chondrocyte activity in OA, such as apoptosis and autophagy. PDK1 has been identified as an upstream activator of PI3K/AKT. Upon PDK1 activation, downstream PI3K/AKT phosphorylation affects the expression of Bax/Bcl-2 and Casp-3, mediating apoptosis (42). Using the small molecule inhibitor BX795, which pharmacologically targets the specific reduction of PDK1 expression, we observed a reduction in the phosphorylation level of the PI3K/AKT signaling pathway and significant downregulation of autophagy-related (ATG7, LC3-I/II, and Beclin-1) and apoptosis-related (Bax, Bcl-2, and Casp-3) genes in chondrocytes (**Figures 7A**, **8**). These results revealed that PDK1 can be used as a diagnostic marker for OA. Additionally, PDK1 is involved in inflammation-mediated autophagy and apoptosis in chondrocytes through the PI3K/AKT and MAPK signaling pathways.

In summary, we found that PDK1 is a signature gene for OA and might provide insights into its early diagnosis and clinical treatment. The chondroprotective effect by target-specific PDK1 inhibition is mediated by the repression of MAPK and PI3K/AKT signaling axes. The mechanisms include attenuation of LPS-induced inflammation levels and apoptosis while repairing autophagy in OA cartilage dysregulation. Although our findings are preliminary, we provided a new potential target for OA treatment and therapy. Nevertheless, further validation and promotion of its applications in clinical studies are needed.

## Data availability statement

Publicly available datasets were analyzed in this study. This data can be found here: the Gene Expression Omnibus (GEO, https://www.ncbi.nlm.nih.gov/geo/) platform.

## Ethics statement

The animal study was reviewed and approved by Medical Ethics Committee of the First Affiliated Hospital of Guangxi Medical University (Approval number: 2022-E320-01). Written informed consent was obtained from the individual(s) for the publication of any potentially identifiable images or data included in this article.

## Author contributions

JY designed the study and revised the manuscript. JM, HL, and HD conducted data analysis and performed the experiments. JL and JFL wrote the draft of the manuscript. All authors contributed to the article and approved the submitted version.
