# Supplementary material for: Identification of the osteoarthritis signature gene PDK1 by machine learning and its regulatory mechanisms on chondrocyte autophagy and apoptosis
Source: Front Immunol. 2023 Jan 6;13:1072526. doi: 10.3389/fimmu.2022.1072526 (PMC9853447; doi:10.3389/fimmu.2022.1072526)
Supplement: Supplementary file 1 [file DataSheet_1.pdf]

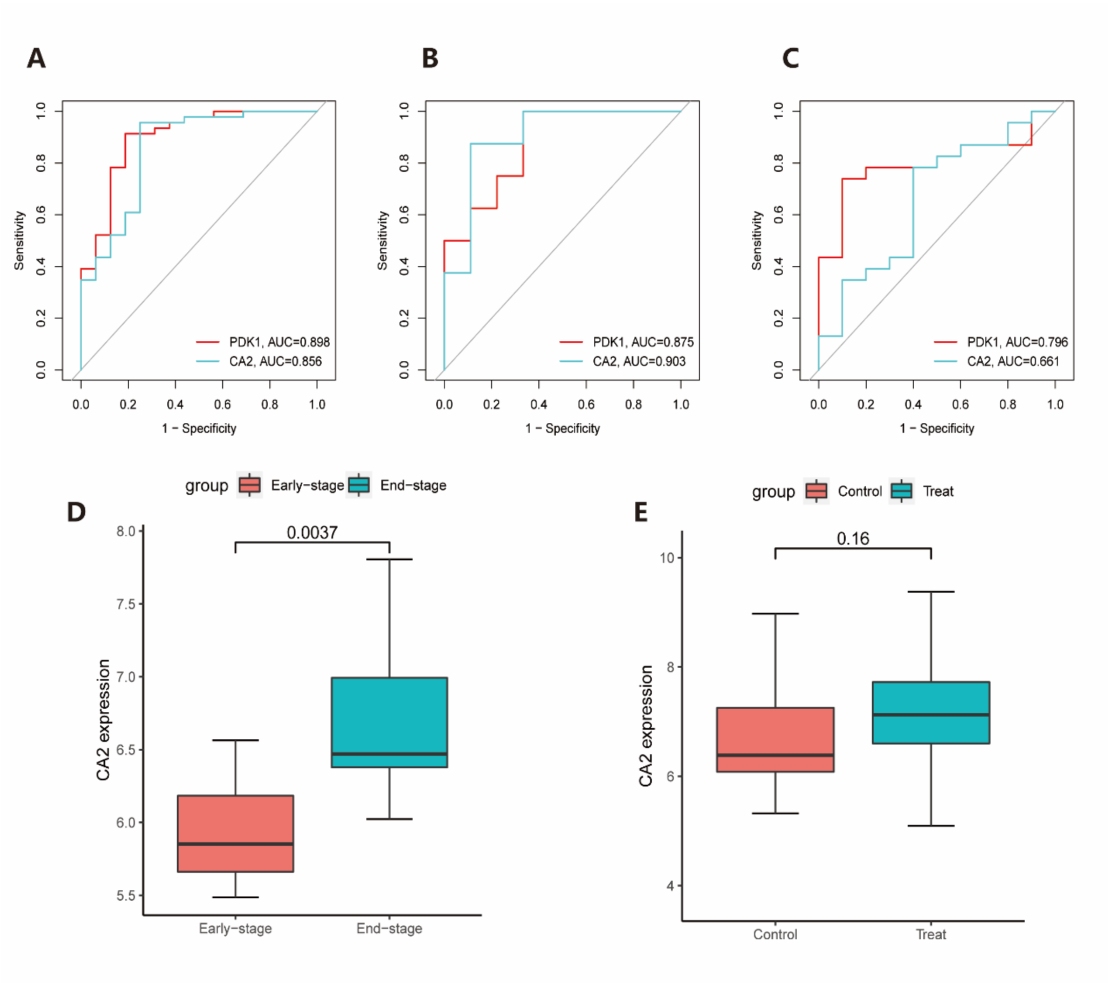

Figure S1: ROC curves and differential analysis for assessing the diagnostic value of signature genes. (A) Training cohort's ROC curves of the feature genes PDK1 and CA2. (B, C) ROC curves for the signature genes PDK1 and CA2 in GSE32317 (B) and GSE55457 (C) validation cohort. (D, E) Expression levels of CA2 gene in the validation cohorts, D: GSE32317; E: GSE55457. A $p < 0.05$ was considered statistically significant.

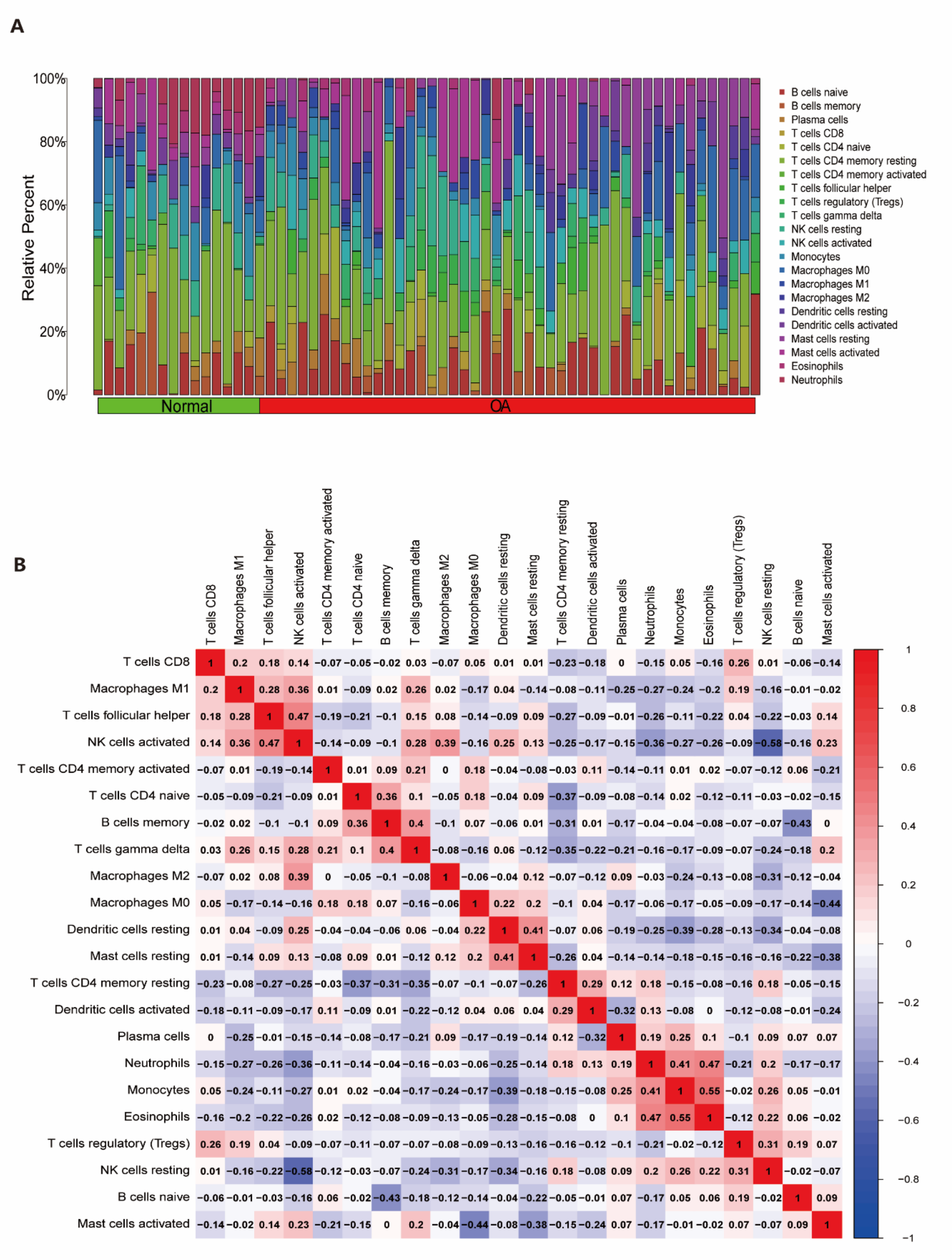

Figure S2: The distribution of 22 immune cell infiltration between normal and OA samples were visualized via the CIBERSORT algorithm. (A) Bar graph of the relative percentages of 22 immune cells between normal and OA samples. The green box at the bottom of the bar represents normal cartilage, the red is OA, and the different colors on

the left represent different types of immune cells. (B) Correlation matrix of 22 immune cells. Red, blue, and white represent high, low, and same correlation, respectively. A $p < 0.05$ was considered statistically significant.

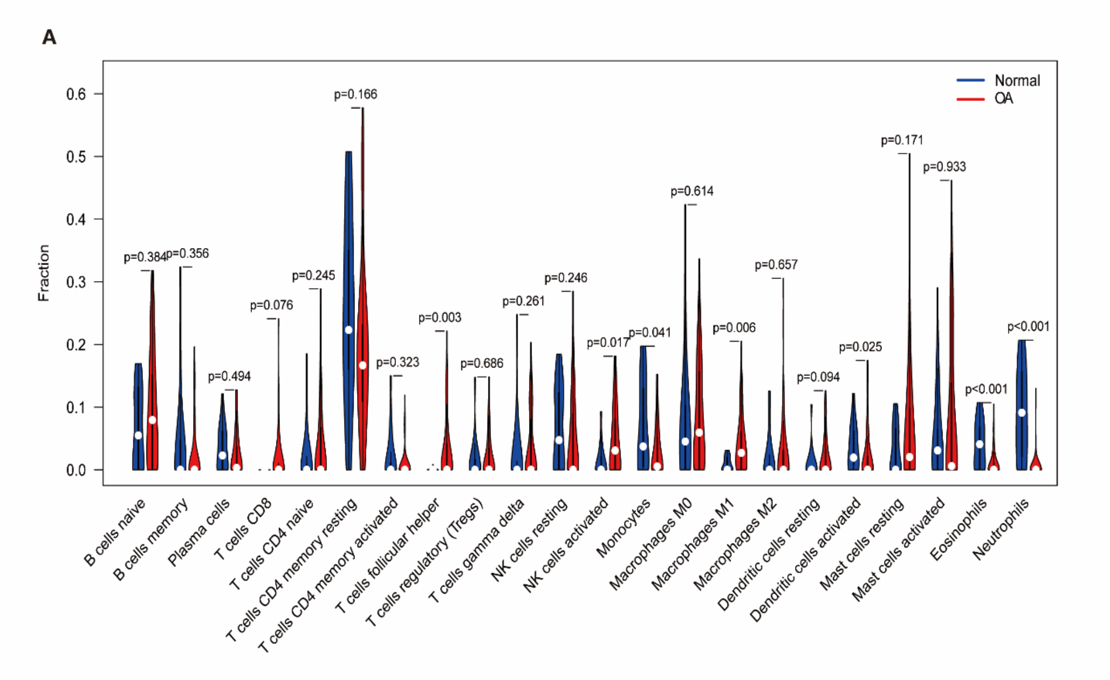

Figure S3: Differential levels of 22 immune cells between normal and OA cartilage, blue represents normal cartilage samples and red represents OA.
